# The effect of proteolytic activity of starter cultures on technologically important properties of yogurt

**DOI:** 10.1002/fsn3.427

**Published:** 2016-09-29

**Authors:** Elahe Amani, Mohammad Hadi Eskandari, Shahram Shekarforoush

**Affiliations:** ^1^ Department of Food Science and TechnologyCollege of AgricultureShiraz UniversityShirazIran; ^2^ Department of Food HygieneSchool of Veterinary MedicineShiraz UniversityShirazIran

**Keywords:** acidifying activity, exopolysaccharide, physicochemical properties, proteolytic activity, starter cultures, yogurt

## Abstract

In this study, the effects of proteolytic activity of yogurt starter bacteria on physicochemical and technological properties of yogurt were investigated. Moreover, impact of proteolytic activity and production of exopolysaccharide (EPS) on the performance of each strain were screened. In order to compare the textural properties of yogurt samples, four parameters were evaluated: syneresis, water‐holding capacity, cohesiveness, and hardness. Results showed that strains with high proteolytic activity had lower acidifying activity during fermentation and storage. Samples containing EPS‐producing starter cultures had low proteolytic activity except samples K, L, and M. These differences related to nature and characteristics of each strain. Counts of starter cultures in samples produced using strains with high proteolytic activity were higher than other samples. Textural analysis data showed significant differences (*p* < .05) among strains in the four tested parameters. Strains with high proteolytic activity showed lower texture properties than other samples. Evaluation of sensory characteristics also showed samples prepared using strains with low or medium proteolytic activity and produced with EPS‐producing strains have higher overall acceptability than other samples. Accordingly, microbial, physicochemical, and sensory properties of produced yogurts confirm that proteolytic activity is one of the most effective factors in quality of product and performance of each strain.

## 1 Introduction

Organoleptic characteristics of yogurt are affected by several factors, including the type of milk, microbiological quality, the technology used in making the yogurt, and others. However, the starter culture has outstanding role in technological and organoleptic characteristics in fermented product. Lactic acid bacteria have different technological properties; one of the important technological properties is proteolytic activity that is needed for growth of bacteria in milk and has impact on properties of fermented product. Lactic acid bacteria (LAB) are characterized by their high demand for essential growth factors such as peptides and amino acids. However, milk does not contain sufficient free amino acids and peptides to allow growth of LAB (Abu‐Tarboush, 1996). Therefore, these microorganisms use their own proteolytic activity, a complex system of proteinases and peptidases, which enable them to use milk casein as a source of amino acids and nitrogen. The essential substrate for such proteolysis is casein; however, limited degradation of whey proteins may also occur. Resulting of this slight activity is a breakdown of only 1–2% of milk protein (Belkaaloul, Chekroun, Ait‐Abdessalam, Saidi, & Kheroua, 2010). Due to proteolytic nature of *Lactobacillus delbrueckii* ssp. *bulgaricus* that leads to production of essential amino acids and because of symbiotic relationship of *L. delbrueckii* ssp. *bulgaricus* and *S. thermophilus*, these bacteria are able to grow in yogurt (Shihata & Shah, 2000). Therefore, type of strains and ratio of the two organisms used for inoculation are effective on degree of proteolysis in yogurt. These properties of starter cultures have been linked to its importance for texture, taste, and flavor development during fermentation and storage period. During storage period, yogurt texture changes due to degradation of the protein network. It also contributes directly to taste and flavor by the formation of peptides and free amino acids as well as by liberation of such substrates for further catabolic changes and thereby formation of volatile flavor compounds. In terms of taste, proteolytic activity causes bilateral effect; peptides can taste bitter or delicious and amino acids can taste sweet, bitter, or broth‐like (Zainoldin & Baba, 2012).

The study by Ramchandran and Shah (2009) showed that proteolytic activity has adverse effect on textural properties of yogurt. Other researches also indicate that yogurts containing EPS‐ producing starter cultures have higher proteolytic activity (Peterson, Dave, McMahon, Oberg, & Broadbent, 2000; Ramchandran & Shah, 2010). Ramchandran and Shah (2010) stated that, proteolytic activity has improving effect on the survival of the starter cultures in the product. Slocum et al., (1988) investigated that proteolytic activity of yogurt culture influences the keeping quality; therefore, it is better to minimize proteolysis during the production and storage of yogurt.

With regard to the effect of proteolytic activity of yogurt cultures on the quality during storage and other technological properties of yogurt, one of the important aims in this study was to determine the proteolytic activity of yogurt cultures using the *o*‐pthaldialdehyde‐based spectrophotometric method. Correlation of this characteristic with other technological properties of yogurts also was evaluated. Moreover, effect of proteolysis on performance of starter cultures during fermentation and storage were estimated.

## 2 Material and Methods

Agar and d‐glucose were purchased from Merck (Merck, Darmstadt, Germany). Skimmed milk powder, whole milk powder, and cream powder were produced by Pegah Fars Company (Shiraz, Iran). Sodium chloride, phenol red, crystal violet, safranin, acetone, M17 agar, MRS agar, phenolphthalein, trichloroacetic acid, ethanol 98%, *o*‐phthaldialdehyde, hydrochloric acid, sulfuric acid, ß‐mercaptoethanol, sodium tetraborate, sodium dodecyl sulfate, and sodium hydroxide were from purchased from Merck.

### 2.1 Preparation of inoculums

The strains used in this study were isolated from traditional Iranian yogurt samples. These strains were isolated and identified by Rushanzadeh (2011) in Shiraz University, Iran.

They were identified as *Lactobacillus delbrueckii subsp. bulgaricus and Streptococcus thermophilus* on the basis of morphological, biochemical, and genotypic characteristics. The isolated bacteria were activated from their frozen forms (stored in 40 g 100 per mL glycerol at −80°C) by culturing them in De Man, Rogosa and Sharpe (MRS) broth (Merck, Darmstadt, Germany) for *L. bulgaricus* and *S. thermophilus* in M17 broth (Merck, Darmstadt, Germany); then incubated at 37°C for 24 hr. Thereafter, they were subcultured in MRS agar and were incubated anaerobically using Gas Pack system (Merck Anaerocult type A, Germany) at 37°C for 72 hr for *L. bulgaricus*, and in M17 agar and incubated at 37°C for 48 hr in aerobic condition for *S. thermophilus* (IDF 1997, 2003). For inoculation into milk, these were grown in specific medium broth until they reached late exponential growth phase (approximately, 10^8^ cfu/ml overnight). The cells were harvested by centrifugation at (SW14R, Froilabo, Paris, France) 10,000*g* for 10 min at 4°C. The pellet was then washed twice with sterile distilled water. The resultant pellet was suspended in entire milk used for yogurt production (Lim, Suntornsuk, & Suntornsuk, 2009). For production control sample, we used Sacco 480 starter culture (blends of EPS^+^ *S. thermophilus* and *L. bulgaricus*).

### 2.2 Yogurt making

Yogurt mixes were made using mixtures of skim milk powder and cream powder (Pegah Fars Dairy Co., Iran) to prepare reconstituted milk (14% w/w), then homogenized and stored at refrigerator (4°C) overnight. The following day, it was pasteurized at 90°C for 5 min under agitation in a water bath followed by cooling to 45°C. The heating and cooling processes were carried out in a closed container to minimize losses due to evaporation. This was followed by inoculation with different strains of *S. thermophilus* (1 × 10^8^ cfu/ml) and *L. bulgaricus* (1 × 10^8^ cfu/ml). The inoculated milk was then mixed thoroughly and dispensed in 100 mL polystyrene cups, sealed with aluminum sheet, and incubated at 42°C until the pH dropped to 4.6 ± 0.1. The fermentation was stopped by transferring the samples immediately to refrigerator maintained at 4°C ± 1. The samples were kept there and at 7‐day intervals (up to 28 days), were subjected for further use. Characteristics of isolates have been shown in (Table 1).

**Table 1:** Culture combinations used for manufacture of experimental yogurts

Sample^a^	*L. delbrueckii* ssp. *bulgaricus*	*S. thermophilus*
A (control)	–	–
B	88s^b^	3w^b^
C	88s	5
D	88s	6
E	122	3w
F	122	5
G	122	6
H	109	3w
I	109	5
J	109	6
K	110p	3w
L	110p	5
M	110p	6

a All culture combinations consisted of equal amounts of *Streptococcus thermophilus* and a *Lactobacillus delbrueckii ssp. bulgaricus* culture.

b Numbers refer to Native Culture Collection Numbering.

### 2.3 Methods of analysis

#### 2.3.1 Acidification activity of strains

During fermentation, acid‐producing activities of all batches were recorded at 1 hr intervals at 40°C using a pH meter (model ST 300; Ohaus, Singapore, USA). All pH measurements were performed in triplicate (Ramchandran & Shah, 2010).

#### 2.3.2 Postacidification and titratable Acidity analysis

The postacidification was determined as pH after 1, 7, 14, 21, and 28 days of storage using a pH meter (do Espírito Santo, Perego, Converti, & Oliveira, 2012). Results were expressed as degree Dornic (Fadela, Abderrahim, & Ahmed, 2009).

#### 2.3.3 Microbiological analysis

During the cold storage, starter culture plate counts were determined at 1, 7, 14, 21, and 28 days. *S. thermuphilus* colonies were enumerated in M17 agar after incubation aerobically at 37°C for 48 hr (IDF 1997, 2003); while *L. bulgaricus* were counted on MRS agar after incubation anaerobically at 37°C for 72 hr (IDF 1997, 2003). The results were obtained as the logarithms of the number of colony forming units per mL (log cfu/ml) of yogurt. The microbiological analyses were performed in two replicates.

#### 2.3.4 Determination of crude EPS content

Method of Ramchandran and Shah (2010) used for determination of crude extractable EPS quantity, involving protein and EPS precipitation and the EPS finally collected by centrifuging at 2000*g* at 4°C for 15 min. The crude EPS was dried in a hot air oven (40°C) until two consecutive weights exhibited a difference of <0.001 g. The results were expressed as milligrams of dried crude of EPS per 100 g of yogurt (Ramchandran & Shah, 2010).

#### 2.3.5 Evaluation of the proteolysis

pH of inoculated samples before incubation were reduced to 4.6 with glacial acetic acid followed by centrifugation at 4000*g* for 30 min at 4°C. The supernatants were filtered through 0.45 μm syringe membrane filter (Merck, Darmstadt, Germany). The serum of the experimental samples at days 1, 7, 14, 21, and 28 of storage were also centrifuged and filtered similar to the above. The filtered solutions were stored at −20°C until assayed (1–2 weeks).

Free amino acid content in filtrated samples represent the extent of proteolysis measuring by the *o*‐phthaldialdehyde (OPA) at 340 nm within 2 min using spectrophotometer (UV 9200, Raylight, Beijing, China). The absorbance of the inoculated samples (before incubation) were deducted from the corresponding absorbance of yogurt samples to obtain the amount of free amino acids released as a consequence of the proteolytic activity of the starter cultures during fermentation and storage (Ramchandran & Shah, 2010).

#### 2.3.6 Changes in spontaneous whey separation

Set‐style yogurt samples were prepared in conical centrifuge tubes (25 g). Then, syneresis was evaluated by centrifuging at 500*g* for 10 min at 4°C. Syneresis is expressed as the weight percentage of serum released by centrifugation (Matumoto‐Pintro, Rabiey, Robitaille, & Britten, 2011).

#### 2.3.7 Measurement of water‐holding capacity

Samples (50 g) from each batch were weighed in centrifuge tubes and incubated at 40°C, after which the set gels were stored during storage period at 4°C. The tubes were centrifuged at 3000*g* for 10 min at 4°C. The whey was separated, then weighed and results expressed as the weight percentage of serum released by centrifugation (Riener, Noci, Cronin, Morgan, & Lyng, 2009).

#### 2.3.8 Texture evaluation

For texture analysis, the yogurt samples were kept in polystyrene cups (45 mm diameter) of 80 ml (4 ± 1°C) for 28 days. Textural properties was determined by means of a texture analyzer—TA‐XT2 (CT3 4500, Brookfield, Middleboro, UK). Determination of the texture profile for each sample was done by measuring the force of penetration of a probe in the sample at a well‐defined speed. A cylindrical probe (SMSP/25) (2.54 cm in diameter and 3.81 cm in height) was used, with crosshead speed of the probe and the penetration depth of 2.0 mm/s and 15 mm, respectively. For each force–time curve, obtained by two successive compressions of each sample, the following texture profile parameters were determined: the maximum force (in g) recorded during the first compression as a measure of sample hardness; the ratio (dimensionless) of the area under the force curve measured during the second compression to the one measured during the first compression as a measure of cohesiveness. Each yogurt sample was tested in three replicate (Gauche, Tomazi, Barreto, Ogliari, & Bordignon‐Luiz, 2009).

#### 2.3.9 Sensory analysis

A number of 15 trained panelists who consume yogurts regularly in their diets and have previous experience in taste evaluation were selected to rate sensory properties of yogurt. Yogurt samples were organoleptically examined according to the method modified from Bayarri, Carbonell, Barrios, and Costell (2011) with scores between 1 and 9 (1 = dislike extremely, and 9 = like extremely) for flavor, body and texture, appearance, and color. Panelists evaluated all yogurt samples after storage for 7 days at 4°C.

#### 2.3.10 Statistical analysis

The majority of experiments were performed in triplicate. All data were analyzed by one‐way analysis of variance (ANOVA) followed by the Duncan's multiple range test. Statistical significance (*p *<* *.05) was assessed using the SAS 9.1 software (SAS Institute, North Carolina), Pearson correlation test was also employed. Nonparametric data were analyzed using Kruskal–Wallis test. *p* values <.05 were considered statistically significant. Analysis was performed using a SPSS package (SPSS 16 for windows, SPSS Inc, Chicago, IL).

## 3 Results and Discussion

### 3.1 Acidifying kinetics during incubation

**Figure 1:**
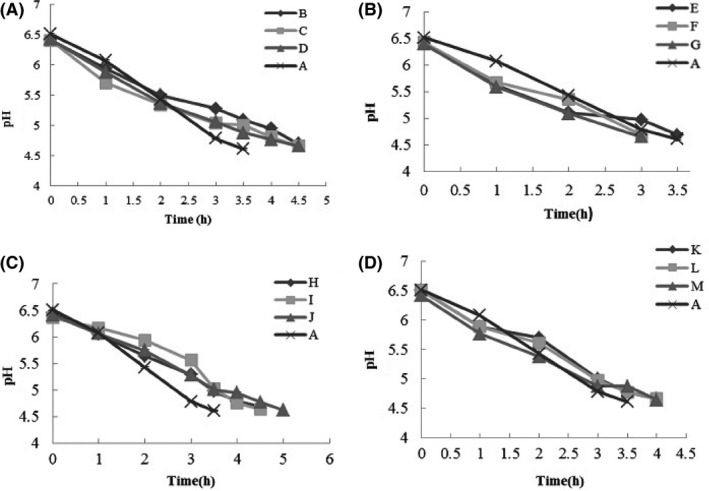
Figures represent reduction in pH of yogurts made using native starter cultures during yogurt fermentation. (A): *Lactobacillus bulgaricus* strain 88s, (B): *L. bulgaricus* strain 122, (C): *L. bulgaricus* strain 109, (D): *L. bulgaricus* strain 110p. In all samples, each strain of *L. bulgaricus* are in combination with three strains of *S. thermophilus* (3w, 5, 6). For details of samples see Table 1

Figure 1 shows typical pH profiles with incubation time, as they were obtained from continuous pH measurement during the yogurt fermentation process. As could be expected on the basis of the chemical acidification reaction that underlies the fermentation process, pH dropped during the 3–5 hr to values 4.6 in all experiments. *L. delbrueckii* ssp. *bulgaricus* strains 88s and 122 in combination with the three strains of* S. thermophilus* have more ability in acid production and showed fastest Acidifying kinetics. The final level of lactic acid, which is the main product of the metabolic activity of starter cultures, as well as the acidification rate during yogurt production, depends on the strains and their associations (Béal, Skokanova, Latrille, Martin, & Corrieu, 1999). Moreover, it was observed that samples produced by strains with low proteolytic activity had sigmoidal pH decrease but starter cultures with high proteolytic activity showed different acidification profiles and fermentation times had been longer. For example, *L. delbrueckii* ssp. *bulgaricus* strains 109 and 110p in combination with the three strains of *S. thermophilus* showed the highest proteolytic activity with low acidification activity. These results confirm that isolates showing the highest acidifying activity need not necessarily have the most proteolytic activity, as reported by other authors (Durlu‐Ozkaya, Xanthopoulos, Tinail, & Litopoulou‐Tzanetaki, 2001; Nieto‐Arribas, Poveda, Seseña, Palop, & Cabezas, 2009). Actually, nature and characteristics of each strain led to different ability; therefore, some strains with high proteolytic activity do not have ability for degradation of lactose and consumption of its products (glucose and galactose that produced by breaking of lactose) for lactic acid production. According to other studies, different factor could be effective on acid‐producing ability such as proteolysis and lactose metabolism and amino acids (Çeilik, 2007; Özer & Atasoy, 2002).

### 3.2 Postacidification and titratable acidity

The results of postacidification (pH) and titratable acidity during the shelf‐life of the yogurts are presented in Table 2. After 28 days of cold storage, pH dropped to pH 4.07–4.38 and titratable acidity varied from 109 to 126 g/100 g. These acid‐production trends during storage are similar to other research (Çeilik, 2007). However, some studies showed this trend because of producing some metabolites become reversed (Ramchandran & Shah, 2009). The highest acidification activity was observed in samples made by *L. delbrueckii* ssp. *bulgaricus* strains 88s and 122 in combination with three strains of *S. thermophilus*. *Lactobacillus. bulgaricus* strains 109 and 110p with high proteolytic activity exhibited lower ability to produce acid during storage at 4°C for 28 days (pH = 4.32–38 and acidity = 109–117). Sample M (*L. bulgaricus* strains 110p in combination with *S. thermophilus* strains 6) showed lowest ability to produce acid and had pH = 4.57 and acidity, 82.35 at first and reached to pH = 4.39 and acidity, 109.80 after 28 days. Strains 109 and 110p of *L. bulgaricus* with high proteolytic activity exhibited higher ability to produce acid during storage at 4°C for 28 days. The changes in pH and acidity during storage were found to be similar in samples with similar proteolytic activity. Changes of acidity in control sample were low (pH = 4.58–4.42 and acidity = 83.25–111.15).

**Table 2:** Change in pH and titratable acidity (Dornic) in yogurts prepared by different combination of starter cultures during storage at 4°C

Treatment	pH					Acidity				
	Storage period (day)					Storage period (day)				
	1	7	14	21	28	1	7	14	21	28
A (control)	4.58^aA^ ± 0.00	4.50^aB^ ± 0.00	4.46^aC^ ± 0.00	4.44^aD^ ± 0.00	4.42^aDC^ ± 0.00	83.25^aC^ ± 1.38	101.25^efgB^ ± 0.87	108.90^fgA^ ± 1.27	111.15^ghA^ ± 0.63	111.20^ghA^ ± 1.90
B	4.53^cA^ ± 0.02	4.29^hiB^ ± 0.01	4.18^iC^ ± 0.02	4.17^ghC^ ± 0.02	4.15^hiC^ ± 0.00	72.45^cD^ ± 0.63	104.40^cdeC^ ± 1.27	110.25^efgB^ ± 1.90	116.10^edA^ ± 1.27	118.80^bcdA^ ± 1.27
C	4.53^aA^ ± 0.02	4.23^jB^ ± 0.02	4.19^iC^ ± 0.00	4.15^hD^ ± 0.00	4.11^jE^ ± 0.00	82.35^aD^ ± 1.90	107.10^bcC^ ± 1.27	115.65^bcdB^ ± 1.90	120.15^bcA^ ± 0.63	121.05^bcA^ ± 0.63
D	4.54^aA^ ± 0.02	4.36^efB^ ± 0.02	4.27^ghC^ ± 0.00	4.24^efDC^ ± 0.02	4.20^fgD^ ± 0.00	83.25^aC^ ± 1.23	100.8^efgB^ ± 2.54	122.05^defA^ ± 0.63	115.65^deA^ ± 1.90	117^cdA^ ± 1.27
E	4.55^aA^ ± 0.01	4.32^ghB^ ± 0.01	4.21^iC^ ± 0.00	4.15^hD^ ± 0.00	4.14^ijD^ ± 0.01	81.45^aE^ ± 0.73	101.25^efgD^ ± 1.90	108^gC^ ± 0.13	116.50^deB^ ± 0.63	120.80^bcdA^ ± 2.54
F	4.53^abA^ ± 0.00	4.26^ijB^ ± 0.01	4.08^jC^ ± 0.01	4.08^iC^ ± 0.00	4.07^kC^ ± 0.00	78.75^abC^ ± 2.06	108^bB^ ± 0.77	123.75^aA^ ± 0.63	124.40^aA^ ± 0.34	126.45^aA^ ± 1.26
G	4.55^aA^ ± 0.01	4.24^jB^ ± 0.01	4.21^iC^ ± 0.00	4.19^ghC^ ± 0.01	4.07^kD^ ± 0.00	80.01^aD^ ± 1.27	113.85^aC^ ± 0.73	118.35^bB^ ± 0.89	120.15^bcB^ ± 0.93	122.85^bA^ ± 1.02
H	4.54^dA^ ± 0.00	4.41^cB^ ± 0.02	4.33^efC^ ± 0.03	4.30^dC^ ± 0.00	4.23^eD^ ± 0.02	95.85^cB^ ± 1.27	108.45^hA^ ± 0.65	109.80^fgA^ ± 0.63	110.25^hA^ ± 2.54	117.85^efgA^ ± 1.90
I	4.51^cdA^ ± 0.01	4.37^deB^ ± 0.02	4.37^fgB^ ± 0.03	4.35^cdeB^ ± 0.04	4.32^dB^ ± 0.01	99.90^cdC^ ± 1.27	108.90^fgB^ ± 2.54	110.50^fgAB^ ± 1.81	112.20^cdA^ ± 1.90	113.45^fA^ ± 0.55
J	4.55^abA^ ± 0.00	4.41^cB^ ± 0.00	4.30^fgD^ ± 0.00	4.35^cD^ ± 0.00	4.31^dD^ ± 0.01	75.78^abD^ ± 2.08	99.45^fghC^ ± 0.5	108.81^fgB^ ± 1.40	112.05^fghA^ ± 0.70	113.85^fgA^ ± 0.41
K	4.54^bcA^ ± 0.01	4.39^cdeB^ ± 0.00	4.38^bcdB^ ± 0.00	4.26^edB^ ± 0.02	4.36^bcC^ ± 0.01	74.25^bcC^ ± 1.11	98.10^ghB^ ± 1.29	110.25^bcdeA^ ± 1.90	114.85^defA^ ± 1.10	110.25^ghA^ ± 0.93
L	4.52^cdA^ ± 0.04	4.55^iB^ ± 0.00	4.42^bC^ ± 0.02	4.41^abC^ ± 0.01	4.38^bC^ ± 0.00	81^cdD^ ± 0.45	96.75^ibB^ ± 1.59	103.95^hB^ ± 0.31	105.30^iAB^ ± 0.52	109.35^hA^ ± 0.95
M	4.57^cdA^ ± 0.00	4.49^aB^ ± 0.01	4.41^bcC^ ± 0.01	4.39^bcC^ ± 0.01	4.33^cdD^ ± 0.02	82.35^cdC^ ± 1.80	98.10^ibB^ ± 1.54	99.90^hB^ ± 0.57	102.11^jB^ ± 1.05	109.80^hA^ ± 1.60

Presented values are the means of three replicate trials (±*SD*). For details of samples, see Table 1. Means in the same column with different lowercase alphabets are significantly different (*p *<* *.05) for each type of yogurt. Means in the same row with different uppercase alphabets are significantly different (*p *<* *.05) for a particular day of storage.

### 3.3 Changes in the counts of yogurt bacteria

The changes in the viable counts (log cfu/ml) of *S. thermophilus* and *L. bulgaricus* during refrigerated storage of yogurt are presented in Table 3. During the shelf‐life, counts of two starters were stable and ranged, as an average, from 7.30 to 9.55 log cfu/ml. Although there are different standards for count of starters, acceptable count is about 10^7^ cfu/ml (Ramchandran & Shah, 2010). This confirms that the native starter cultures isolated from indigenous yogurt remained viable in the product until the end of storage (28 days) which is satisfactory for the yogurt production. In general, the counts of strains with high proteolytic activity (109 and 110p of *L. bulgaricus* in combination with three strains of *S. thermophilus*) were higher (*p *<* *.05) than others. In fact, continued proteolysis (Table 4) improve survival of the starters in the product by providing the essential growth factors in the form of peptides and amino acids (Ramchandran & Shah, 2010). In all samples, counts of two starters showed a reduction at day 14, 21 or at end of the storage, but counts of starter cultures remained stable in yogurts made using starter with high proteolytic activity throughout the storage period. This confirms the protective effect of proteolysis on survival of the starter cultures. Another possible reason could be attributed to slightly lower pH and higher acidity (Table 5) in the yogurts made from strains with higher proteolytic activity. The counts of *L. delbrueckii* ssp*. bulgaricus* in all samples except control sample were higher than *S. thermophilus*; mainly because *S. thermophilus* strains are more sensitive to acidic conditions than *L. delbrueckii* ssp. *bulgaricus* strains. In Birollo, Reinheimer, and Vinderola (2000) study; numbers of *S. thermophilus* were higher than the *L. delbrueckii* ssp. *bulgaricus* during storage but Irkin and Eren (2008) reported that the counts of *L. delbrueckii* ssp. *bulgaricus* were higher than the *S. thermophilus* during storage.

**Table 3:** Changes in counts of *S. thermophilus* and *L. delbrueckii* ssp. *bulgaricus* (log CFU/mL) of the yogurts prepared by different combination of local starter cultures during storage at 4°C

Treatment	*S. thermophilus*					*L. delbrueckii ssp. bulgaricus*				
	Storage period (day)					Storage period (day)				
	1	7	14	21	28	1	7	14	21	28
A (control)	9.55^abA^ ± 0.85	9.09^abcdA^ ± 0.19	9.38^aA^ ± 0.12	8.29^deB^ ± 0.21	9.37^aA^ ± 0.47	9.44^abcdAB^ ± 0.01	9.75^abA^ ± 0.32	8.31^eC^ ± 0.23	8.60^bBCc^ ± 0.34	7.38^fD^ ± 0.12
B	9.28^abcA^ ± 0.02	9.03^abcdA^ ± 0.46	8.82^abcdA^ ± 0.31	8.64^bcdA^ ± 0.36	7.80^deB^ ± 0.28	9.29^abcdA^ ± 0.02	9.08^bcdA^ ± 0.08	8.99^Aabcd^ ± 0.25	9.35^aA^ ± 0.24	8.47^cdA^ ± 1.10
C	8.44^efgA^ ± 0.56	7.91^eA^ ± 0.10	7.92^efA^ ± 0.16	8.16^defB^ ± 0.45	7.81^deA^ ± 0.11	9.20^abcdA^ ± 0.07	8.96^cdA^ ± 29/0	8.10^eB^ ± 0.04	8.98^abA^ ± 0	7.60^efB^ ± 0.42
D	8.63^cdefgAB^ ± 0	8.10^cdeBC^ ± 0	8.47^bcdeA^ ± 0.26	9.06^abcA^ ± 0.08	7.34^eD^ ± 0.36	9.82^aA^ ± 0.09	8.73^cdC^ ± 0.05	8.73^abcdeB^ ± 0.09	9.21^aA^ ± 0.25	7.40^fD^ ± 0.14
E	8.66^cdefgB^ ± 0.26	8.50^cdeB^ ± 0.28	9.89^abcA^ ± 0.07	9.16^abA^ ± 0.90	8.40^cdB^ ± 0.14	9.18^abcdA^ ± 0.48	9.06^bcdA^ ± 0.78	8.88^abcdeA^ ± 0.41	9.08^abA^ ± 0.30	8.74^bcdA^ ± 0.58
F	9.16^abcdA^ ± 0.43	8.50^cdeAB^ ± 0.28	8.17^defB^ ± 0	8.78^abcdAB^ ± 0.16	8.95^abcA^ ± 0.26	8.82^abcdA^ ± 0.34	8.72^cdA^ ± 0.45	8.66^bcdeA^ ± 0.61	8.94^abA^ ± 14/0	8.77^bcdA^ ± 0.24
G	7.98^gA^ ± 0.13	7.95^eA^ ± 0.18	7.90^gA^ ± 0	8.47^cdeA^ ± 0.67	8.38^cdA^ ± 0.12	8.65^cdAB^ ± 0.14	8.69^cdAB^ ± 0.15	8.51^deB^ ± 0.04	8.52^bcB^ ± 0.25	8.88^bcdA^ ± 0.24
H	9.73^aA^ ± 0.56	9.66^aAB^ ± 0.37	8.74^abcdC^ ± 0.22	8.56^cdC^ ± 0.40	9.96^abcBC^ ± 0.87	9.10^abcdAB^ ± 0.58	10.00^aA^ ± 0.23	9.11^abcdAB^ ± 0.34	8.92^abB^ ± 0.26	9.09^abAB^ ± 0.23
I	9.16^abcdA^ ± 0.37	9.52^abA^ ± 0.31	9.18^aA^ ± 0.05	9.31^aA^ ± 0.01	9.00^abcA^ ± 0.91	9.11^abcdA^ ± 0.31	9.41^abcA^ ± 0.65	9.22^abcA^ ± 0.71	9.20^aA^ ± 0.03	9.19^abA^ ± 0.56
J	9.67^aA^ ± 0.56	9.19^abcdA^ ± 0.24	9.32^aAB^ ± 0.70	9.05^abcA^ ± 0.30	9.20^aA^ ± 0.06	9.70^abA^ ± 0.60	9.43^abcA^ ± 0.58	9.38^aA^ ± 0.60	7.69 ^dB^ ± 0.90	8.16^deB^ ± 0.22
K	8.40^fgB^ ± 0.06	9.25^abcA^ ± 0.34	9.38^aA^ ± 0.24	9.20^abA^ ± 0.22	9.27^aA^ ± 0.19	9.47^abcdA^ ± 0.08	9.06^bcdB^ ± 0.92	9.38^aAB^ ± 0.46	9.36^aAB^ ± 0.33	9.27^aAB^ ± 0.24
L	8.91^bcdefB^ ± 0.18	9.55^abA^ ± 0.42	9.06^abAB^ ± 0.02	9.02^abcAB^ ± 017	8.77^abB^ ± 0.16	9.59^abcA^ ± 0.46	9.41^abcA^ ± 0.60	8.99^abcdA^ ± 0.43	8.84^abA^ ± 0.35	8.60^bcdA^ ± 0.49
M	9.29^abcA^ ± 0.55	8.96^bcdA^ ± 0.28	9.16^aA^ ± 0.59	8.74^abcdA^ ± 0.20	8.61^abcdA^ ± 0	9.26^abcdA^ ± 0.89	8.98^cdA^ ± 0.44	8.99^abcdA^ ± 0.12	8.79^abA^ ± 0.23	9.20^aA^ ± 0.38

Presented values are the means of three replicate trials (±*SD*). For details of samples, see Table 1. Means in the same column with different lowercase alphabets are significantly different (*p *<* *.05) for each type of yogurt. Means in the same row with different uppercase alphabets are significantly different (*p *<* *.05) for a particular day of storage.

**Table 4:** Changes in EPS content (g/100 g) during storage of the yogurts prepared by different combination of local starter cultures during storage at 4°C

Treatment	Storage period (day)				
	1	7	14	21	28
A (control)	47.20^aA^ ± 5.09	26.00^cB^ ± 7.48	17.20^dceB^ ± 3.95	12.70^efB^ ± 7.90	12.20^efgB^ ± 5.93
B	13.20^cdB^ ± 2.82	12.80^edfB^ ± 3.17	16.40^cdeB^ ± 1.13	15.06^efA^ ± 2.54	50.20^aA^ ± 5.93
C	8.53^cdB^ ± 0.84	13.20^edfB^ ± 1.69	20.00^dceA^ ± 4.52	11.00^efB^ ± 1.97	6.80^fgB^ ± 2.11
D	44.40^aA^ ± 2.26	18.33^cdefB^ ± 5.77	15.30^cdeB^ ± 2.40	16.80^deB^ ± 3.93	10.00^efgB^ ± 4.09
E	49.20^aA^ ± 5.09	37.60^bB^ ± 2.26	25.00^bcC^ ± 3.39	13.40^efD^ ± 4.24	10.00^efgD^ ± 1.28
F	41.00^aA^ ± 4.24	14.93^cdefB^ ± 3.95	13.00^edB^ ± 6.48	13.13^efB^ ± 3.93	9.40^efgB^ ± 1.97
G	8.88^cdC^ ± 1.28	20.73^cdeB^ ± 4.24	34.40^bA^ ± 3.11	12.85^efC^ ± 4.24	12.75^defgC^ ± 3.47
H	6.50^dD^ ± 2.01	15.00^cdefDC^ ± 1.41	25.50^bcBA^ ± 4.36	34.00^cA^ ± 1.41	22.50^cdBC^ ± 3.10
I	6.25^dC^ ± 1.06	9.40^efC^ ± 2.28	10.00^eBC^ ± 2.54	26.20^cdA^ ± 5.61	22.60^cdBA^ ± 3.80
J	9.00^cdB^ ± 1.90	14.20^efdB^ ± 2.82	34.17^bA^ ± 7.43	5.10^fB^ ± 2.10	4.70^gB^ ± 2.40
K	31.60^bA^ ± 3.39	18.30^cdefB^ ± 1.97	14.60^cdeBC^ ± 3.67	11.70^efBC^ ± 5.93	8.30^fgC^ ± 1.98
L	9.26^cdB^ ± 4.92	12.80^edfB^ ± 2.22	51.60^aA^ ± 5.50	18.53^deB^ ± 4.34	15.10^defB^ ± 9.10
M	42.60^aA^ ± 4.24	22.66^cdB^ ± 12.21	15.60^dceB^ ± 1.41	14.40^efB^ ± 2.26	10.50^efgB^ ± 3.95

Presented values are the means of three replicate trials (±*SD*). For details of samples, see Table 1. Means in the same column with different lowercase alphabets are significantly different (*p *<* *.05) for each type of yogurt. Means in the same row with different uppercase alphabets are significantly different (*p *<* *.05) for a particular day of storage.

**Table 5:** Changes in extent of proteolysis (A_340_) in the yogurts prepared by different combination of local starter cultures during storage at 4°C

Treatment	Storage period (day)				
	1	7	14	21	28
A (control)	0.18^kB^ ± 0.01	0.27^lA^ ± 0.06	0.27^lA^ ± 0.03	0.28^kA^ ± 0.01	0.29^gA^ ± 0.09
B	0.44^efE^ ± 0.06	0.51^efgB^ ± 0.01	0.57^gC^ ± 0.01	0.64^hiB^ ± 0.07	0.81^dA^ ± 0.04
C	0.50^bcE^ ± 0.02	0.60^bcD^ ± 006	0.70^cC^ ± 0.09	0.77^cdB^ ± 0.07	0.87^cA^ ± 0.03
D	0.35^jlE^ ± 0.07	0.43^hD^ ± 0.04	0.51^hC^ ± 0.05	0.58^jB^ ± 0.04	0.62^fA^ ± 0.08
E	0.32^jE^ ± 0.08	0.49^fgD^ ± 0.04	0.62^efC^ ± 0.01	0.69^fgB^ ± 0.06	0.80^dA^ ± 0.04
F	0.37^hiE^ ± 0.07	0.57^cdD^ ± 0.08	0.64^deC^ ± 0.03	0.70^efB^ ± 0.13	0.78^dA^ ± 0.10
G	0.33^jE^ ± 0.05	0.43^hD^ ± 0.07	0.50^hC^ ± 0.02	0.61^jlB^ ± 0.09	0.71^eA^ ± 0.02
H	0.54^aE^ ± 0.02	0.62^abD^ ± 0.01	0.83^Ac^ ± 0.03	0.94^aB^ ± 0.02	1.05^aA^ ± 0.05
I	0.38^cdE^ ± 0.04	0.48^bcD^ ± 0.07	0.66^dcC^ ± 0.08	0.73^deB^ ± 0.06	0.98^bA^ ± 0.04
J	0.46^edE^ ± 0.06	0.55^deD^ ± 0.04	0.66^cdC^ ± 0.05	0.55^deD^ ± 0.03	0.87^cA^ ± 0.03
K	0.41^ghE^ ± 0.05	0.49^gD^ ± 0.07	0.58^fgC^ ± 0.04	0.69^fgB^ ± 0.06	0.74^eA^ ± 0.02
L	0.42^fgE^ ± 0.02	0.57^cdD^ ± 0.01	0.75^bC^ ± 0.09	0.89^bB^ ± 0.02	1.00^bA^ ± 0.05
M	0.41^ghE^ ± 0.04	0.55^deD^ ± 0.03	0.69^cC^ ± 0.04	0.88^bB^ ± 0.03	1.00^bA^ ± 0.04

Presented values are the means of three replicate trials (±*SD*). For details of samples, see Table 1. Means in the same column with different lowercase alphabets are significantly different (*p *<* *.05) for each type of yogurt. Means in the same row with different uppercase alphabets are significantly different (*p *<* *.05) for a particular day of storage.

### 3.4 Changes in crude EPS concentration

The concentration of crude extractable EPS at various intervals of storage are shown in Table 4. Initially, in most samples, the concentration of EPS increased, and after that a decreasing trend was observed except in samples B and F which showed steady increase until 28th day of storage. Moreover, concentration of EPS in other samples such as E, F, K, and M decreased during storage. The decrease in EPS content during storage could be attributed to the presence of enzymes capable of degrading EPS (Degeest, Mozzi, & De Vuyst, 2002; Purwandari et al., 2007). Indeed, some strains for their survival and viability should use EPS‐degrading enzymes for producing sugar as a source of energy. In sample B, EPS content increased during the 28 days of storage (*p *<* *.05). Amatayakul, Halmos, Sherkat, and Shah (2006) have also found that concentration of EPS in yogurt made using ropy starter cultures increased during storage.

They also have stated that this was not found in yogurt made using capsular EPS‐producing starter cultures, in which the EPS concentration remained constant during storage at 4°C.

Variations in the method of estimating the EPS, differences in the types of EPS, as well as strain variations could be the possible reasons for the differences observed (Ramchandran & Shah, 2010). The concentration of EPS in yogurts made using EPS starter cultures ranged from 30 to 60 mg/100 g. The amount of extracted curd EPS in Amatayakul et al. (2006) were also in these same ranges. It is interesting to note that a low concentration of EPS (10–20 mg/100 g) was found in yogurt produced with non‐EPS‐producing starter cultures. The low amount of EPS, detected in the yogurt made with non‐EPS‐producing starter cultures, might be due to the residue of lactose remaining after the purification (Amatayakul et al., 2006). According to the results, EPS‐producing strains have lower proteolytic activity. For example, *L. bulgaricus* strains 109 was non‐EPS‐producing starter cultures and showed high proteolytic activity. Also, *L. bulgaricus* strains 88s and 122 were EPS‐producing starter and showed lower proteolytic activity. Nevertheless, EPS‐producing starter cultures such as *L. bulgaricus* strains 110p and 96 in combination with three strains of *S. thermophilus* showed higher proteolytic activity than other EPS‐producing starter cultures. Peterson et al. (2000) reported that EPS^+^ starter culture has more proteolytic activity.

### 3.5 Changes in extent of proteolysis

The results shown in Table 5 indicate that there were significant differences (*p *<* *.05) among the values of proteolytic activity for strains. Over the storage period of 28 days, all yogurts exhibited continued increase in extent of proteolysis. Similar observation was reported by Ramchandran and Shah (2010). Significant differences (*p *<* *.05) in proteolytic activity values were found between strains. Overall, the increase in level of free amino acids during storage was higher for *L. bulgaricus* strains 109 and 110p in combination with the three strains of *S. thermophilus*. These strains have higher proteolytic activity with *S. thermophilus* strains 3w and 5 and reached up to 1–1.05 during storage. Samples produced with *L. bulgaricus* strains 122 in combination with the three strains of *S. thermophilus* had low proteolytic activity. Sample D have lower proteolytic activity than other samples (0.35–0.62 units).

### 3.6 Whey separation and water‐holding capacity

The percentage of spontaneous whey separation and water‐holding capacity of samples is given in Table 6. Syneresis of all samples except samples H, K, L, and M decreased during of storage; However, syneresis in samples H, K, L, and M decreased until 21 days of storage, then increased in the last week. La Torre, Tamime, and Muir (2003) reported that reduction in syneresis during of storage related to metabolic activity of starters and reduction in pressure of protein network. Ramchandran and Shah (2009) reported syneresis decreased until 14th day, indicating a rapid recovery of structure after the destruction of structure, however after 14 days this trend reversed and syneresis increased that represents the disintegration of the structure during storage. Actually, the sudden increase in syneresis is related to high proteolytic activity of isolates; therefore, leading to disintegration of the structure during storage. Water‐holding capacity of all samples except samples H, K, L, and M increased during storage. Samples made using high proteolytic activity (*L. bulgaricus* strains 109 and 110p in combination with the three strains of *S. thermophilus*) showed highest syneresis and lowest water‐holding capacity. In addition, samples made using *L. bulgaricus* strains 88s in combination with the three strains of *S. thermophilus* showed lowest syneresis (6.31–5.33% on the first day and decreased to 2.71–3.25% during 28 days of storage). These samples also showed highest water‐holding capacity (63.50–67.11% on the first day and increased to 73.19–77.60% during 28 days of storage). Therefore, these results confirm that yogurts prepared using cultures with low proteolytic activity had better water‐holding capacity and thereby lower syneresis.

**Table 6:** Change in spontaneous whey separation and water holding capacity (%, w/w) in yogurts prepared by different combination of starter culture during storage at 4°C

Treatment	Synersis					WHC				
	Storage period (day)					Storage period (day)				
	1	7	14	21	28	1	7	14	21	28
A (control)	6.03^cdefA^ ± 0.07	5.16^cdefB^ ± 0.32	4.98^bcB^ ± 0.25	4.16^abcC^ ± 0.13	3.43^efghD^ ± 0.09	64.45^deD^ ± 0.18	67.59^fgC^ ± 0.12	74.16^aB^ ± 0.59	75.33^abB^ ± 0.82	78.80^aA^ ± 0.30
B	5.33^fA^ ± 0.71	4.86^defAB^ ± 0.29	4.91^bcdAB^ ± 0.15	3.96^bcdeBC^ ± 0.50	3.25^ghiC^ ± 0.04	66.93^bC^ ± 1.11	67.07^fgC^ ± 0.26	68.89^defB^ ± 0.64	72.31^efA^ ± 0.48	73.19^edA^ ± 0.49
C	6.31^cA^ ± 0.25	6.24^bcA^ ± 0.23	5.17^bB^ ± 0.07	3.15^gC^ ± 0.47	2.71^jC^ ± 0.19	67.11^bE^ ± 0.16	69.89^cdD^ ± 0.10	72.24^abcC^ ± 0.39	74.11^cdB^ ± 0.23	75.82^bcA^ ± 0.61
D	5.43^efA^ ± 0.07	4.90^defAB^ ± 0.35	4.10^defghBC^ ± 0.65	3.40^defgDC^ ± 0.27	2.73^jD^ ± 0.28	63.50^eD^ ± 0.97	66.70^fghC^ ± 0.96	72.97^abB^ ± 0.76	76.25^aA^ ± 0.40	77.60^abA^ ± 1.44
E	5.33^fA^ ± 0.34	4.23^fB^ ± 0.15	3.74^ghC^ ± 0.16	3.23^fgD^ ± 0.34	3.10^hijD^ ± 0.15	65.13^cdD^ ± 0.33	71.27^bC^ ± 0.29	72.53^abB^ ± 0.52	74.29^bcA^ ± 0.46	74.41^defA^ ± 0.60
F	6.39^cA^ ± 0.22	5.99^bcdeAB^ ± 0.28	5.34^bB^ ± 0.15	4.34^abcC^ ± 0.35	3.86^deC^ ± 0.36	65.60^bcdD^ ± 0.47	66.71^fghC^ ± 0.46	68.18^efgB^ ± 0.12	71.19^gA^ ± 0.35	71.44^efA^ ± 0.11
G	6.50^cA^ ± 0.35	5.15^cdefB^ ± 0.23	4.86^bcdeB^ ± 0.07	4.07^bcdC^ ± 0.36	6.50^cA^ ± 0.25	63.54^eC^ ± 0.45	64.52^ijC^ ± 0.44	66.43^ghB^ ± 0.40	67.60^iB^ ± 0.56	69.87^ghA^ ± 0.50
H	6.48^cA^ ± 0.07	6.10^bcdeA^ ± 0.21	3.93^fghC^ ± 0.35	4.81^aB^ ± 0.51	6.41^bA^ ± 0.15	67.11^bD^ ± 0.23	68.08^efC^ ± 0.40	69.51^cdefB^ ± 0.20	71.78^fgA^ ± 0.29	63.84^iE^ ± 0.16
I	6.23^cdeA^ ± 0.44	7.51^bcdAB^ ± 0.30	5.24^bB^ ± 0.37	4.11^bcC^ ± 0.34	3.37^fghiC^ ± 0.31	61.53^fE^ ± 0.17	63.54^kD^ ± 0.14	64.56^hiC^ ± 0.63	67.84^iB^ ± 0.38	69.02^ghA^ ± 0.33
J	9.28^aA^ ± 0.08	8.09^aB^ ± 0.19	6.69^aC^ ± 0.38	4.26^abcD^ ± 0.12	3.69^defE^ ± 0.14	60.78^fD^ ± 0.36	63.74^jkC^ ± 0.34	64.30^iC^ ± 0.12	69.17^hB^ ± 0.40	71.42^efA^ ± 0.60
K	5.47^defA^ ± 0.20	5.31^cdefA^ ± 0.17	3.98^fghB^ ± 0.06	3.86^cdefB^ ± 0.06	5.13^cA^ ± 0.12	70.00^aC^ ± 0.49	70.57^bcC^ ± 0.39	72.96^abB^ ± 0.59	74.33^bcA^ ± 0.50	70.24^fgC^ ± 0.11
L	5.45^defB^ ± 0.53	4.18^defB^ ± 0.31	4.74^bcdefBC^ ± 0.32	4.17^abcC^ ± 0.24	7.18^aA^ ± 0.05	66.22^bcD^ ± 0.30	67.63^efgC^ ± 0.57	70.99^cdeB^ ± 0.46	72.13^efgA^ ± 0.11	67.69^hC^ ± 0.30
M	5.19^fA^ ± 0.10	4.77^efA^ ± 0.29	4.04^efghB^ ± 0.14	3.92^bcdeB^ ± 0.02	4.83^cdA^ ± 0.35	64.95^cdeC^ ± 0.43	65.74^hiBC^ ± 0.71	66.68^gB^ ± 0.48	69.15^hA^ ± 0.16	67.30^hA^ ± 0.68

Presented values are the means of three replicate trials (±*SD*). For details of samples, see Table 1. Means in the same column with different lowercase alphabets are significantly different (*p *<* *.05) for each type of yogurt. Means in the same row with different uppercase alphabets are significantly different (*p *<* *.05) for a particular day of storage.

### 3.7 Texture analysis

In order to compare the textural properties of the samples, tow parameters were evaluated: hardness and cohesiveness. Texture profile analysis results of the experiments are shown in Table 7. As can be observed in this table, the yogurts made using native strains displayed better textural properties. Analysis variance of textural data showed that there were significant differences (*p *<* *.05) among strains for the two tested parameters. Textural studies conducted by many authors suggested that syneresis, texture, and viscosity of fermented milks were affected by milk composition and type of culture (Chammas, Saliba, Corrieu, & Béal, 2006; Marshall & Rawson, 1999). Since milk composition was kept constant in this study, the differences observed within the cultures were due to the strains.

**Table 7:** Texture profile analysis of yogurts prepared by different combination of starter cultures during storage at 4°C

Treatment	Hardness (g)					Cohesiveness				
	Storage period (day)					Storage period (day)				
	1	7	14	21	28	1	7	14	21	28
A (control)	124.83^bcC^ ± 4.31	128.00^eC^ ± 3.00	128.75^efghBC^ ± 6.01	135.00^deB^ ± 4.94	149.75^bcdA^ ± 0.35	0.40^bcdA^* ± 0.01	0.38^abA^ ± 0.03	0.36^eA^ ± 0.05	0.37^eA^ ± 0.02	0.37^cdA^ ± 0.02
B	127.25^bC^ ± 1.06	141.38^abcdB^ ± 3.18	146.00^abcAB^ ± 6.48	148.50^bcAB^ ± 4.49	153.00^bcA^ ± 4.24	0.35^dB^ ± 0.02	0.37^abAB^ ± 0.02	0.40^abcA^ ± 0.02	0.41^abA^ ± 0.01	0.42^abcA^ ± 0.01
C	155.00^aB^ ± 5.08	148.33^abB^ ± 5.50	149.50^abB^ ± 3.04	156.75^abB^ ± 4.01	181.25^aA^ ± 1.76	0.40^bcdA^ ± 0.03	0.38^abB^ ± 0.01	0.40^abcdAB^ ± 0.01	0.40^abA^ ± 0.01	0.40^bcdA^ ± 0.01
D	107.50^edC^ ± 2.12	151.00^aB^ ± 4.49	156.00^aAB^ ± 2.47	157.33^aAB^ ± 1.41	160.67^bA^ ± 6.36	0.37^cdB^ ± 0.40	0.38^abAB^ ± 0.39	0.40^abcdAB^ ± 0.39	0.41^bAB^ ± 0.08	0.44^abcdA^ ± 0.37
E	129.66^bB^ ± 4.04	133.00^deB^ ± 2.82	135.00^cdefAB^ ±1.70	136.50^deAB^ ± 1.80	144.25^cdefA^ ± 2.42	0.39^cdA^ ± 0.03	0.39^abA^ ± 0.01	0.41^abcA^ ± 0.03	0.39^abA^ ± 0.01	0.39^abcdA^ ± 0.04
F	122.50^bcC^ ± 5.63	134.16^abcBC^ ± 2.70	142.16^bcdAB^ ± 6.60	145.50^defAB^ ± 4.25	151.50^bcAB^ ± 4.30	0.41^abcA^ ± 0.04	0.41^aA^ ± 0.02	0.40^abcA^ ± 0.03	0.40^abA^ ± 0.01	0.41^abcdA^ ± 0.01
G	98.33^fgC^ ± 5.90	108.83^fghiB^ ± 6.22	108.50^jkB^ ± 5.77	128.86^efgA^ ± 4.76	134.16^efgA^ ± 4.25	0.42^abcA^ ± 0.01	0.42^aAB^ ± 0.04	0.42^aAB^ ± 0.02	0.41^bA^ ± 0.03	0.41^abcdA^ ± 0.04
H	97.66^efgC^ ± 2.51	101.33^hiBC^ ± 3.21	112.00^ijkAB^ ± 2.64	121.00^gAB^ ± 3.60	132.00^fgA^ ± 2.82	0.40^bcdA^ ± 0.02	0.39^abAB^ ± 0.02	0.38^deAB^ ± 0.04	0.37^cB^ ± 0.01	0.35^dC^ ± 0.02
I	105.50^eC^ ± 0.70	137.00^cdeBC^ ± 1.41	141.00^bcdeABC^ ±2.82	141.50^cdAB^ ± 1.83	143.50^cdefA^ ± 2.73	0.38^cdA^ ± 0.03	0.40^abA^ ± 0.01	0.38^cdeA^ ± 0.02	0.39^abA^ ± 0.03	0.40^abcdA^ ± 0.03
J	100.00^efC^ ± 3.56	131.00^cdB^ ± 2.64	135.00^cdefB^ ± 3.60	143.50^cdA^ ± 2.12	144.67^efgA^ ± 4.72	0.41^abcA^ ± 0.02	0.40^abAB^ ± 0.02	0.41^abcdAB^ ± 0.01	0.37^cB^ ± 0.02	0.36^cdB^ ± 0.03
K	101.25^eB^ ± 2.76	111.00^fghAB^ ± 3.53	117.83^hijA^ ± 4.28	121.00^gA^ ± 5.54	118.16^hiA^ ± 2.02	0.41^abcA^ ± 0.02	0.40^abA^ ± 0.04	0.40^abcdA^ ± 0.03	0.40^abA^ ± 0.02	0.40^abcdA^ ± 0.02
L	116.50^cdA^ ± 4.04	130.00^eA^ ± 4.24	131.33^defgA^ ± 6.11	136.75^deA^ ± 6.71	117.50^jiB^ ± 6.70	0.41^abcA^ ± 0.02	0.41^aAB^ ± 0.01	0.39^abcdAB^ ± 0.02	0.41^abAB^ ± 0.03	0.36^cdB^ ± 0.01
M	82.16^gB^ ± 4.19	99.66^iA^ ± 3.21	105.00^kA^ ± 1.03	105.75^hA^ ± 1.06	107.16^jA^ ± 3.81	0.41^abcdA^ ± 0.01	0.40^aA^ ± 0.01	0.39^abcdA^ ± 0.03	3.39^abA^ ± 0.03	0.38^abcdA^ ± 0.02

Presented values are the means of three replicate trials (±*SD*). For details of samples, see Table 1.^abcdef^Means in the same column with different lowercase alphabets are significantly different (*p *<* *.05) for each type of yogurt. Means in the same row with different uppercase alphabets are significantly different (*p *<* *.05) for a particular day of storage.

Hardness of all samples except samples K and L increased during storage (*p *<* *.05), but in samples K and L, it increased until 21st day of storage then decreased in the last week. Samples produced by strains with low proteolytic activity showed more hardness than other samples. For example, samples made using *L. bulgaricus* 88s in combination with three strains of *S. thermophilus* had higher hardness than others (*p *<* *.05). Among all samples, hardness of sample C was higher than others and reached 181.25 g at the end of storage. Hardness in samples made using high proteolytic activity (*L. bulgaricus* strains 109 and 110p in combination with three strains of *S. thermophilus*) was lower than other samples.

There was no significant variation (*p *>* *.05) in the cohesiveness of samples throughout the storage period; nevertheless, cohesiveness in samples I, K, and M decreased during storage from 0.40 to 0.41 on the first day of cold storage and reached to 0.35–0.36 at the end of storage.

Overall among all samples, samples made using strains with higher proteolytic activity showed lower textural characteristics than others. Buffa, Morais, Jiménez‐Belenguer, Hernández‐Giménez, and Guamis (2005) also reported that high proteolytic strains are not always the most suitable for use as starter cultures, since excessive proteolysis can cause uncontrolled production of bitter peptides and other undesirable compounds, or even excessive casein hydrolysis resulting in a too‐soft final product. However, Ruas‐Madiedo, Alting, and Zoon (2005) asserted that proteolytic activity of the strains did not seem to play any significant role.

### 3.8 Sensory evaluation

**Figure 2:**
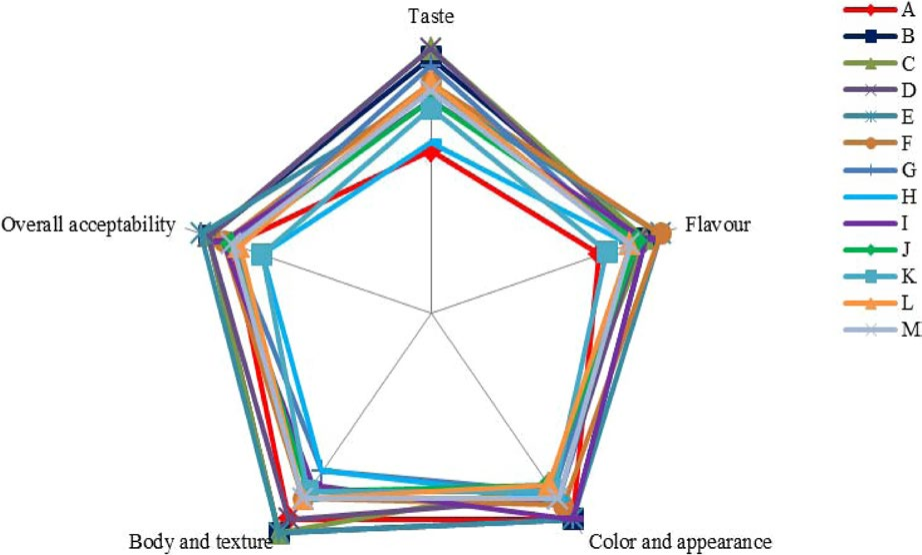
Sensory evaluation values of yogurts after 7 days of storage in 4°C. Means (*n* = 13). For details of samples, see Table 1

Figure 2 presents the sensory evaluation values of the samples. There were different scores, but without significant difference (*p *>* *.05) after storage for 7 days. Samples produced using strains with high proteolytic activity had lower score of flavor, taste, and textural properties than others samples. EPY in terms of sensory characteristics were similar with NEPY but had better textural properties. In general, overall acceptability of EPY prepared using strains with low or medium proteolytic activity was higher than that for NEPY and strains with high proteolytic activity.

### 3.9 Correlation between proteolytic activity and technological parameters

The statistical relationship among proteolytic activity and technological parameters of acidification activity, textural properties, and content of exopolysaccharide were examined using Pearson's correlation procedure, for each combination of bacterial species. Result of correlations is shown in Table 8. Proteolytic activity was correlated to pH (*r* = −.87 to 0.96) and acidity (*r* = .80 to .96). In this study, there was also a correlation between the proteolytic activity and texture properties such as syneresis (*r* = −.65 to .94), water‐holding capacity (0.84 to 0.98), and hardness (0.76 to 0.97). According to the results of the correlation analysis, proteolytic activity of strains is not correlated well with the EPS content, viable counts of two starter cultures and cohesiveness.

**Table 8:** Correlation between proteolysis and technological parameters of yogurts

Treatment	Technological parameters								
	pH	Acidity	St	Lb	EPS	Syneresis	WHC	Hardness	Cohesiveness
A (control)	0.11 NS	0.02 NS	−0.13 NS	−0.40 NS	0.20 NS	0.17 NS	−0.26 NS	0.24 NS	−0.19 NS
B	−0.77**	0.79**	−0.90**	−0.71*	0.86**	−0.89**	0.90**	0.85**	0.71**
C	−0.89**	0.90**	0.51 NS	−0.72*	0.13 NS	−0.85**	0.87**	−0.10 NS	0.05 NS
D	−0.94**	0.92**	−0.37 NS	−0.72*	−0.86**	−0.91**	0.96**	0.76**	0.67*
E	−0.90**	0.93**	0.20 NS	0.34 NS	−0.96**	−0.93**	0.88**	0.74*	−0.23 NS
F	−0.88**	0.89**	−0.15 NS	0.04 NS	−0.84**	−0.90**	0.96**	0.73*	−0.16 NS
G	−0.92**	0.89**	0.62 NS	0.26 NS	0.26 NS	−0.94**	0.88**	0.83**	0.16 NS
H	−0.95**	0.87**	−0.75*	−0.41 NS	0.78**	0.65*	0.87**	0.97**	0.64*
I	−0.78**	0.82**	−0.42 NS	−0.06 NS	0.74*	−0.94**	0.98**	0.38 NS	0.24 NS
J	−0.81**	0.89**	−0.75*	−0.80**	−0.26 NS	−0.99**	0.98**	0.72*	0.69*
K	−0.80**	0.85**	0.64*	−0.07 NS	−0.90**	−0.01 NS	−0.26 NS	0.87**	−0.02 NS
L	−0.90**	0.97**	−0.03 NS	−0.56 NS	0.06 NS	0.44 NS	0.90**	0.10 NS	−0.56 NS
M	−0.94**	0.96**	−0.54 NS	−0.24 NS	−0.83**	0.29 NS	0.54 NS	0.82**	−0.87**

**correlation is significant at the 0.01 level

*correlation is significant at the 0.05 level

NS. not significant

Presented values are the correlation between proteolysis and technological parameters of yogurts prepared by different combination of starter cultures during storage at 4°C. For details of samples, see Table 1.

## 4 Conclusion

The findings of this study illustrates the considerable difference among technological characteristics of yogurts made from each native strains. All isolates showed medium or high acidifying activity during incubation and storage at 4°C. During shelf‐life, counts of two starter cultures were stable and ranged, as an average, from 7.30 to 9.55 log cfu/ml. Strains were EPS‐producing culture and with low or medium proteolytic activity such as *L. bulgaricus* strains 88s and 122 have displayed better textural parameters. These strains also showed low syneresis and high WHC than other strains. Cohesiveness values in the majority of samples displayed no significant difference during storage. The strains with high proteolytic activity showed low acidifying capacity during incubation and storage and counts of these strains were higher. Textural parameters were negatively affected by the high proteolytic activity. Samples containing EPS‐producing starter culture displayed low proteolytic activity except samples K, L, and M showed these strains would be good candidates as starter culture for using in the industrial. However, their potential use depends on further assessment of their aptitude for producing of fermented products in industrial scales and their preservation by freezing and spray drying.

## Conflict of Interest

No potential conflict of interest relevant to this article was reported.
